# Association of smoking and polygenic risk with the incidence of lung cancer: a prospective cohort study

**DOI:** 10.1038/s41416-022-01736-3

**Published:** 2022-02-22

**Authors:** Peidong Zhang, Pei-Liang Chen, Zhi-Hao Li, Ao Zhang, Xi-Ru Zhang, Yu-Jie Zhang, Dan Liu, Chen Mao

**Affiliations:** 1 Department of Epidemiology, School of Public Health, Southern Medical University, Guangzhou, Guangdong China; 2 The Laboratory for Precision Neurosurgery, Nanfang Hospital, Southern Medical University, Guangzhou, Guangdong China; 3 State Key Laboratory of Molecular Neuroscience and Center of Systems Biology and Human Health, Division of Life Science, Hong Kong University of Science and Technology, Hong Kong, China; 4 Microbiome Medicine Center, Department of Laboratory Medicine, Zhujiang Hospital, Southern Medical University, Guangzhou, Guangdong China

**Keywords:** Lung cancer, Risk factors

## Abstract

**Background:**

Genetic variation increases the risk of lung cancer, but the extent to which smoking amplifies this effect remains unknown. Therefore, we aimed to investigate the risk of lung cancer in people with different genetic risks and smoking habits.

**Methods:**

This prospective cohort study included 345,794 European ancestry participants from the UK Biobank and followed up for 7.2 [6.5–7.8] years.

**Results:**

Overall, 26.2% of the participants were former smokers, and 9.8% were current smokers. During follow-up, 1687 (0.49%) participants developed lung cancer. High genetic risk and smoking were independently associated with an increased risk of incident lung cancer. Compared with never-smokers, HR per standard deviation of the PRS increase was 1.16 (95% CI, 1.11–1.22), and HR of heavy smokers (≥40 pack-years) was 17.89 (95% CI, 15.31–20.91). There were no significant interactions between the PRS and the smoking status or pack-years. Population-attributable fraction analysis showed that smoking cessation might prevent 76.4% of new lung cancers.

**Conclusions:**

Both high genetic risk and smoking were independently associated with higher lung cancer risk, but the increased risk of smoking was much more significant than heredity. The combination of traditional risk factors and additional PRS provides realistic application prospects for precise prevention.

## Background

Lung cancer is the most commonly diagnosed cancer and has the highest mortality worldwide among the general population and males, and it has the second leading mortality and the third incidence among females. In 2018, there were more than 2 million new cases and 1.7 million deaths from lung cancer [1]. Tobacco exposure is the leading cause of lung cancer, despite differences in the intensity of smoking and the type of cigarettes, and ~90% of lung cancers are attributed to smoking [2]. In addition, genetic factors also play essential roles in cancer development. Twin studies [3] and heritability estimation based on genome-wide association studies (GWASs) [4, 5] indicated that genetic factors contribute far less to incident lung cancer than environmental factors, including smoking. However, population-based prospective studies of smoking and genetic risk in lung cancer have not been fully validated.

Over the past decade, GWASs have identified multiple susceptibility loci associated with lung cancer risk, including *TP63*, *TERT*, *CDKN2A/B* and *CHRNA3/5* [6–9]. However, while consistently and significantly associated with the lung cancer risk, each common variant’s impact is modest. Aggregating multiple single-nucleotide polymorphisms (SNPs) with tiny functions to generate a composite polygenic risk score (PRS) may explain the genetic risk of complex diseases [10]. In addition, multiple genes, including *CHRNA3/5*, were strongly associated with lung cancer, smoking behaviours [11], and nicotine addiction [12]. Although previous studies have reported a significant association with lung cancer based on case-control designs [13, 14], the relevance of combining these risk scores and smoking for individual subjects and whether smoking and genetic risk have a synergistic effect remains uncertain. Therefore, we hypothesised that smoking and genetic risk are independently associated with incident lung cancer.

This study’s primary purpose was to investigate whether there are differences in the association between smoking and new-onset lung cancer among individuals with low, intermediate or high genetic risk in a large population-based cohort. The second aim was to investigate the possible interaction between genetic risk and smoking for incident lung cancer.

## Methods

### Study design

The UK Biobank study started in 2006 and, until 2010, recruited >500,000 participants aged 40–69 years from the general population at 22 assessment centres throughout the UK [15]. Participants provided information on smoking and other potentially health-related aspects through extensive baseline questionnaires, verbal interviews and physical measurements. Moreover, blood samples were collected for genotyping.

Participants were excluded if they withdrew from the study (*n* = 1298), their genotype data does not meet the quality control conditions, related to another one more than second-degree, or were non-European ancestry (*n* = 44,072). Besides, participants with missing data on smoking or covariates were excluded (*n* = 75,546). Participants with a history of cancer at baseline were also excluded (*n* = 35,814).

### Polygenic risk score

Polygenic risk scores were created following an additive model for previously published common genetic variants associated with lung cancer. To identify relevant risk loci, we began by searching the NHGRI-EBI GWAS Catalog of published GWAS [16]. Then, we reviewed both the original manuscript and supplementary materials to identify SNPs, risk alleles, and effect sizes. SNPs were selected for each locus according to the criteria of independent (*r*^2^ < 0.1), common (minor allele frequencies [MAF] > 0.01 in 1000 Genomes Project European population), UK Biobank available, large sample size in the development cohort, and smallest *P* value. The number of risk alleles (0, 1 or 2) for everyone was summed after multiplication with the effect size between the SNPs and each trait. A total of 33 SNPs from eight studies were used (eTable 1 in the Supplement) [8, 9, 17–22]. This polygenic risk score was then z-standardised based on values for all individuals and categorised into low (lowest quintile), intermediate (quintiles 2–4) and high (highest quintile) risk.

### Smoking status and pack-years

Touchscreen questionnaires collected information on smoking status and pack-years at baseline. Detailed definitions of smoking status and the pack-years of smoking were provided in eTable 2 in the Supplement. All participants were categorised as never, former or current smoking according to their smoking status, and as no (0), light (0.1–19.9), intermediate (20–39.9), or heavy (≥40) smoking according to the pack-years of smoking.

### Outcomes

Participants with incident lung cancer were identified as having a diagnosis in national cancer registries after baseline assessment. Diagnoses were recorded using the International Classification of Diseases-9 (ICD-9) and ICD-10 coding system (eTable 3 in Supplement). Death was ascertained via linkage to death registries. We calculated the follow-up time from the date of attendance to the date of first diagnosis, date of death, March 31, 2016 for Wales and England, and October 31, 2015 for Scotland, whichever occurred first.

### Covariates

All models were adjusted for age, sex, education, socioeconomic status (household income and Townsend deprivation index [23]), body mass index (BMI), physical activity, diet, alcohol consumption, passive smoking, occupational exposure, the relatedness of individuals in the sample and first 20 principal components of ancestry. Body mass index (BMI) (kg/m^2^) was calculated for all UK Biobank participants based on their measured weight and height. Duration and intensity of physical activity were ascertained by touchscreen questionnaires based on the validated International Physical Activity Questionnaire [24]. A healthy diet was calculated based on the Dietary Approaches to Stop Hypertension (DASH) recommendation, associated with multiple cancer types [25, 26]. Alcohol consumption was calculated based on US Dietary Guidelines for Americans 2015–2020 [27]. Exposure to tobacco smoke from others at home or outside for more than an hour per week was considered passive smoking. Occupational exposure is based on self-reported exposure to asbestos, paints, thinners, glues, pesticides, diesel exhaust, or other chemical smog at work.

### Statistical analyses

Baseline characteristics of participants were summarised across incident lung cancer status as a percentage for categorical variables, mean (standard deviation [SD]) for normally distributed variables, and median (interquartile range) for skewed variables. The association between genetic-risk categories, smoking categories, and the combination of genetic and smoking categories (nine categories with low genetic risk and never-smoking as a reference, 12 categories with low genetic risk and no smoking pack-years as a reference) and incident lung cancer were explored using multivariable Cox proportional hazard models. The assumption for proportional hazards was evaluated by tests based on Schoenfeld residuals [28]; violation of this assumption was not observed in our analyses. The area under the curve (AUC) of receiver operating characteristic (ROC) curves was used to assess each model’s predictive ability, including PRS, smoking, and the combination. The associations between PRS and incident lung cancer were evaluated on a continuous scale with restricted cubic spline curves based on multivariable Cox proportional hazards models. Moreover, interactions between polygenic risk scores and smoking status or pack-years were tested. The population-attributable fractions (PAFs), which estimate the proportion of events that would have been prevented if all individuals had been in the never-smoking category, were calculated [29]. The distribution of smoking status in the Health Survey for England (HSE) [30] and European Prospective Investigation into Cancer and Nutrition (EPIC) [31] with better representation to England and the European population were included in the analysis to deal with the incomplete representation of the UK Biobank [32].

Several sensitivity analyses were conducted to verify the robustness of the results. The risk of incident lung cancer was analysed using genetic-risk quintiles and pack-years of smoking in more subdivided groups. The association was also adjusted for self-reported and hospital diagnosed chronic obstructive pulmonary disease (COPD) and chronic pulmonary infections (definitions in eTable 3) at baseline, which may be important confounding factors [33, 34]. The sensitivity analysis excluded participants who had third-degree or higher relatedness to further reduce non-random distribution of risk genes, developed outcomes within the first two years of follow-up to avoid reverse causality, and had a mismatch between calculation and self-reported never-smoking. Moreover, stratified analyses were performed to estimate potential modification effects according to sex (female or male), age (<60 or ≥60 years). Analyses were undertaken using R v3.6.1 (R Center for Statistical Computing, Vienna, Austria). *P* value < 0.05 (two-sided) was considered significant.

## Results

### Participants characteristics

**Fig. 1:**
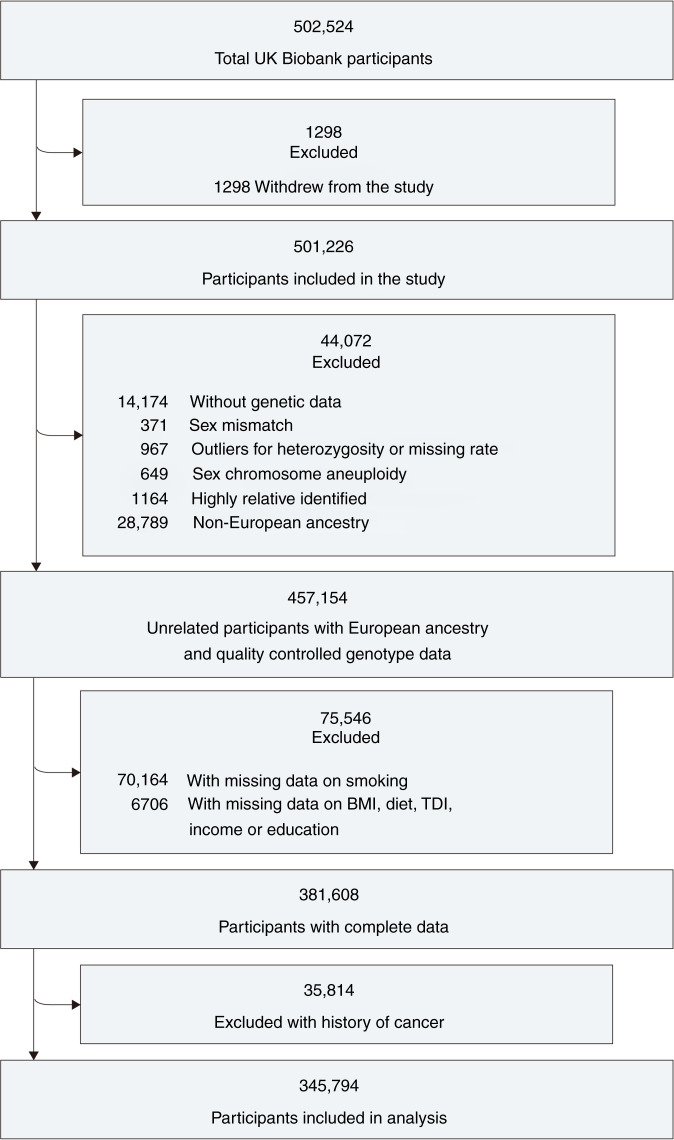
Flow chart of participant enrolment. BMI body mass index, TDI Townsend deprivation index.

A total of 345,794 European individuals with a complete genotype and phenotype were included in the analysis of incident lung cancer, and their detailed information is shown in Fig. 1. Their mean (SD) age was 56.3 (8.0) years, and 186,330 (53.9%) were female. The PRS was normally distributed among all participants (eFigure 1 in Supplement). There were 90,727 (26.2%) former smokers and 33,994 (9.8%) current smokers, among which 40,889 (11.8%) individuals had intermediate smoking exposure (20–39.9 pack-years) and 19,027 (5.5%) individuals had heavy smoking exposure (≥40 pack-years). The participant characteristics are provided in Table 1.

**Table 1:** Baseline characteristics.

Characteristics	No. (%)		
	Overall (*n* = 345,794)	No incident lung cancer (*n* = 344,107)	Incident lung cancer (*n* = 1687)
Age (years), mean (SD)	56.3 (8.0)	56.3 (8.0)	61.6 (5.7)
Sex			
Female	186,330 (53.9)	185,549 (53.9)	781 (46.3)
Male	159,464 (46.1)	158,558 (46.1)	906 (53.7)
Smoking status			
Never	221,073 (63.9)	220,819 (64.2)	254 (15.1)
Former	90,727 (26.2)	90,009 (26.2)	718 (42.6)
Current	33,994 (9.8)	33,279 (9.7)	715 (42.4)
Smoking pack-years:			
No (0)	222,009 (64.2)	221,741 (64.4)	268 (15.9)
Light (0.1–19.9)	63,869 (18.5)	63,632 (18.5)	237 (14.0)
Intermediate (20–39.9)	40,889 (11.8)	40,362 (11.7)	527 (31.2)
Heavy (≥40)	19,027 (5.5)	18,372 (5.3)	655 (38.8)
Body mass index (kg/m^2^):			
Mean (SD)	27.4 (4.8)	27.4 (4.8)	27.4 (4.8)
<18.5	1783 (0.5)	1759 (0.5)	24 (1.4)
18.5–24.9	113,239 (32.7)	112,713 (32.8)	526 (31.2)
25–29.9	146,803 (42.5)	146,096 (42.5)	707 (41.9)
≥30	83,969 (24.3)	83,539 (24.3)	430 (25.5)
Physical activity (min/week)			
Regular physical activity	200,264 (57.9)	199,373 (57.9)	891 (52.8)
Some physical activity	105,802 (30.6)	105,301 (30.6)	501 (29.7)
No regular physical activity	39,728 (11.5)	39,433 (11.5)	295 (17.5)
Diet (DASH score)			
Mean (SD)	22.1 (4.0)	22.1 (4.0)	20.5 (4.5)
Ideal (26–35)	76,627 (22.2)	76,367 (22.2)	260 (15.4)
Intermediate (19–25)	209,760 (60.7)	208,859 (60.7)	901 (53.4)
Poor (7–18)	59,407 (17.2)	58,881 (17.1)	526 (31.2)
Alcohol consumption(g/day)			
Intermediate (0)	82,686 (23.9)	82,190 (23.9)	496 (29.4)
Ideal (male: 0–28; female: 0–14)	170,569 (49.3)	169,925 (49.4)	644 (38.2)
Excessive (male: >28; female: >14)	92,539 (26.8)	91,992 (26.7)	547 (32.4)
Passive smoking			
No	273,496 (79.1)	272,257 (79.1)	1239 (73.4)
Yes	72,298 (20.9)	71,850 (20.9)	448 (26.6)
Occupational exposure			
Rarely/never	270,885 (78.3)	269,283 (78.3)	1602 (95.0)
Sometimes	46,309 (13.4)	46,269 (13.4)	40 (2.4)
Often	28,600 (8.3)	28,555 (8.3)	45 (2.7)
Townsend deprivation index, median (interquartile range)	–2.3 [–3.7, 0.3]	–2.3 [–3.7, 0.2]	–0.7 [–2.9, 2.8]
Household income (£)			
<18,000	77,133 (22.3)	76,426 (22.2)	707 (41.9)
18,000–30,999	87,841 (25.4)	87,335 (25.4)	506 (30.0)
31,000–51,999	91,479 (26.5)	91,187 (26.5)	292 (17.3)
52,000–100,000	70,865 (20.5)	70,721 (20.6)	144 (8.5)
>100,000	18,476 (5.3)	18,438 (5.4)	38 (2.3)
Education			
Lower qualification	179,094 (51.8)	177,920 (51.7)	1174 (69.6)
Higher qualification	166,700 (48.2)	166,187 (48.3)	513 (30.4)
Genetic-risk category			
Low (lowest quintile)	69,155 (20.0)	68,907 (20.0)	248 (14.7)
Intermediate (quintiles 2–4)	207,465 (60.0)	206,446 (60.0)	1019 (60.4)
High (highest quintile)	69,174 (20.0)	68,754 (20.0)	420 (24.9)

*DASH* adjusted Dietary Approaches to Stop Hypertension, *SD* standard deviation.

^a^Body mass index calculated as weight in kilograms divided by height in metres squared.

^b^All variables globally significantly different between groups at *P* < 0.001 except for the mean body mass index.

Over 2,454,915 person-years of follow-up (median [interquartile range] length of follow-up, 7.2 [6.5–7.8] years), there were 1687 cases of incident lung cancer. Participants who developed incident lung cancer were slightly older, more likely to be male, had more smoking exposure, had less physical activity, and had an unhealthy diet. Meanwhile, they also had higher genetic risks.

### Associations of genetic risk with incident lung cancer

**Fig. 2:**
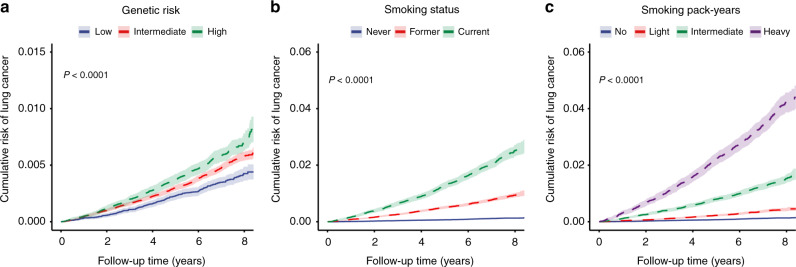
Cumulative risk of incident lung cancer according to genetic risk or smoking. Cumulative risk of incident lung cancer during follow-up according to genetic risk (**a**), smoking status (**b**) and smoking pack-years (**c**).

With the increase in genetic risk, the incidence rate and hazard ratio (HR) of lung cancer gradually increased. After additional adjustment for smoking status or pack-years, the HRs of the high genetic-risk group were 1.73 (95% confidence interval [CI], 1.48–2.02) and 1.69 (95% CI, 1.44–1.97) compared with the low genetic-risk group, and the HRs per SD of PRS increase were 1.16 (95% CI, 1.11–1.22) and 1.16 (95% CI, 1.10–1.21). This result was almost the same as before the adjustment (Table 2). When genetic-risk quintiles were used instead of categories, the same results trend was observed (eTable 4 in Supplement). Figure 2a shows the cumulative risk of incident lung cancer in each genetic-risk group during follow-up.

**Table 2:** Risk of incident lung cancer according to genetic risk.

Genetic risk	Total no. of participants	No. of lung cancer cases (%)	Person-years	IR^a^	Model 1^b^			Model 2^c^			Model 3^d^		
					HR (95% CI)	*P* value	*P* for trend	HR (95% CI)	*P* value	*P* for trend	HR (95% CI)	*P* value	*P* for trend
Low	69,155	248 (0.36)	491,385	0.50	1 (reference)		<0.001	1 (reference)		<0.001	1 (reference)		<0.001
Intermediate	207,465	1019 (0.49)	1,472,759	0.69	1.39 (1.21–1.60)	<0.001		1.40 (1.22–1.61)	<0.001		1.38 (1.20–1.58)	<0.001	
High	69,174	420 (0.61)	490771	0.86	1.73 (1.48–2.03)	<0.001		1.73 (1.48–2.02)	<0.001		1.69 (1.44–1.97)	<0.001	
per SD increase					1.16 (1.11–1.22)	1.41 × 10^−10^	–	1.16 (1.11–1.22)	1.35 × 10^−10^	–	1.16 (1.10–1.21)	9.64 × 10^−10^	–

*IR* incidence rate, *HR* hazard ratio, *CI* confidence interval, *SD* standard deviation.

^a^Incidence rates are provided per 1000 person-years.

^b^Model 1: Cox proportional hazards regression adjusted for age, sex, education, Townsend deprivation index, income, BMI, diet, physical activity, alcohol consumption, occupational exposure, passive smoking, relatedness and first 20 principal components of ancestry; *P* value for trend calculated treating the polygenic risk score as a continuous variable.

^c^Model 2: Cox proportional hazards regression adjusted for Model 1 and smoking status categories; *P* value for trend calculated treating the genetic-risk score as a continuous variable.

^d^Model 3: Cox proportional hazards regression adjusted for Model 1 and smoking pack-years categories; *P* value for trend calculated treating the genetic-risk score as a continuous variable.

### Associations of smoking with incident lung cancer

With the changing smoking status and increasing pack-years, the incidence and HR of lung cancer were also increased. After additional adjustment for PRS, the HRs of the current or heavy smoking group were 14.54 (95% CI, 12.47–16.94) and 17.80 (95% CI, 15.23–20.81), respectively, compared with the never-smoking group. This result was almost the same as before the adjustment (Table 3). When the number of smoking pack-years was given in more subdivided categories, the same trend of results was observed (eTable 5 in Supplement). Figure 2b and c shows the cumulative risk of incident lung cancer in each smoking status and pack-year group during follow-up.

**Table 3:** Risk of incident lung cancer according to smoking categories.

Smoking	Total no. of participants	No. of lung cancer cases (%)	Person-years	IR^a^	Model 1^b^			Model 2^c^		
					HR (95% CI)	*P* value	*P* for trend	HR (95% CI)	*P* value	*P* for trend
Smoking status										
Never smoking	221,073	254 (0.11)	1,574,709	0.16	1 (reference)		<0.001	1 (reference)		<0.001
Former smoking	90,727	718 (0.79)	640,792	1.12	5.33 (4.61–6.17)	<0.001		5.33 (4.61–6.17)	<0.001	
Current smoking	33,994	715 (2.10)	239,414	2.99	14.53 (12.46–16.93)	<0.001		14.54 (12.47–16.94)	<0.001	
Smoking pack-years										
No (0)	222,009	268 (0.12)	1,581,227	0.17	1 (reference)		<0.001	1 (reference)		<0.001
Light (0.1–19.9)	63,869	237 (0.37)	452,690	0.52	3.04 (2.55–3.62)	<0.001		3.04 (2.55–3.63)	<0.001	
Intermediate (20–39.9)	40,889	527 (1.29)	289,086	1.82	8.62 (7.40–10.03)	<0.001		8.61 (7.39–10.02)	<0.001	
Heavy (≥40)	19027	655 (3.44)	131,912	4.97	17.89 (15.31–20.91)	<0.001		17.80 (15.23–20.81)	<0.001	

*IR* incidence rate, *HR* hazard ratio, *CI* confidence interval.

^a^Incidence rates are provided per 1000 person-years.

^b^Model 1: Cox proportional hazards regression adjusted for age, sex, education, Townsend deprivation index, income, BMI, diet, physical activity, alcohol consumption, occupational exposure, passive smoking, relatedness and first 20 principal components of ancestry; *P* value for trend calculated treating each smoking category as continuous variables.

^c^Model 2: Cox proportional hazards regression adjusted for Model 1 and polygenic risk score; *P* value for trend calculated treating each smoking category as continuous variables.

### Associations of smoking and genetic risk with incident lung cancer

**Fig. 3:**
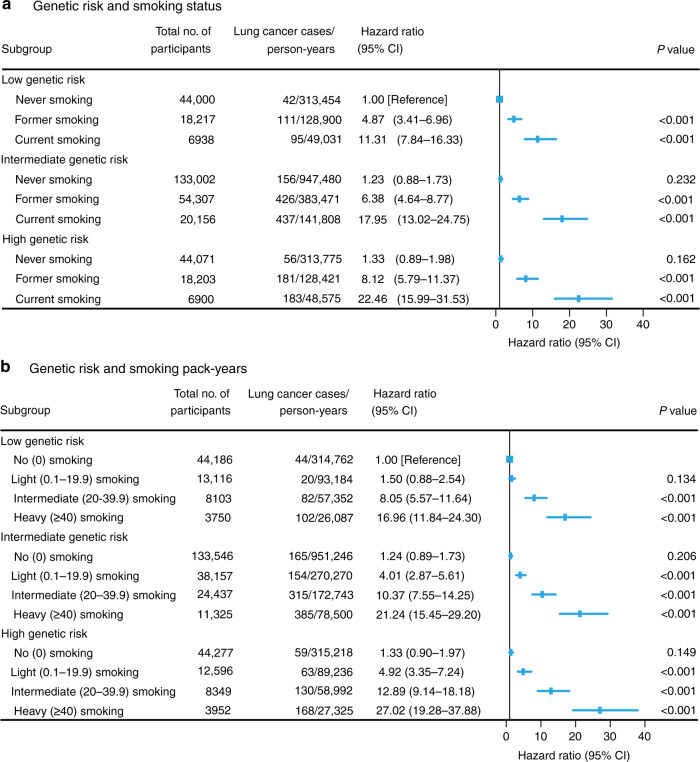
Risk of incident lung cancer according to a combination of genetic risk and smoking. Risk of incident lung cancer according to genetic risk and smoking status (**a**) or genetic risk and smoking pack-years (**b**). The vertical line indicates the reference value of 1.

In each genetic-risk group, the incidence and HR of lung cancer increased with the smoking status deteriorating and pack-years increasing. Compared with the low genetic risk and never-smoking group, there was no significant difference of incident lung cancer risk in the high genetic risk but never-smoking group, while the HR of the low genetic risk but the current smoking group was 11.31 (95% CI, 7.84–16.33). A similar pattern was observed among genetic risk and smoking pack-year groups. The highest risks were observed among individuals with high genetic risk and current smoking (HR, 22.46 [95% CI, 15.99–31.53]) compared with low genetic risk and never-smoking. Individuals with high genetic risk and heavy smoking had a much higher risk of incident lung cancer (HR, 27.02 [95% CI, 19.28–37.88]) compared with those with low genetic risk and no smoking (Fig. 3). There was no significant interaction between the PRS and the smoking status or pack-years (both *P* for interaction > 0.05).

Further analyses stratified by genetic-risk category showed that the association between smoking and lung cancer appeared to increase with increasing genetic risk (Table 4). In the low, intermediate and high genetic-risk groups, the HRs of current smoking were 10.75 (95% CI, 7.28–15.88), 14.86 (95% CI, 12.22–18.07), and 16.85 (95% CI, 12.25–23.19), respectively, compared with never-smoking. Similarly, the HRs of heavy smoking were 16.22 (10.97–23.97), 17.06 (13.97–20.84) and 21.22 (15.34–29.35) compared with no smoking.

**Table 4:** Risk of incident lung cancer according to a smoking category within each genetic-risk category.

Subgroup	Total no. of participants	No. of lung cancer cases (%)	Person-years	IR^a^	HR (95% CI)^b^	*P* value	*P* for trend	*P* for interaction
Genetic risk and smoking status								
Low genetic risk								0.132
Never-smoking	44,000	42 (0.10)	313,454	0.13	1 (reference)		<0.001	
Former smoking	18,217	111 (0.61)	128,900	0.86	4.61 (3.2–6.64)	<0.001		
Current smoking	6938	95 (1.37)	49,031	1.94	10.75 (7.28–15.88)	<0.001		
Intermediate genetic risk								
Never-smoking	133,002	156 (0.12)	947,480	0.16	1 (reference)		<0.001	
Former smoking	54307	426 (0.78)	383,471	1.11	5.22 (4.33–6.29)	<0.001		
Current smoking	20156	437 (2.17)	141,808	3.08	14.86 (12.22–18.07)	<0.001		
High genetic risk								
Never-smoking	44071	56 (0.13)	313,775	0.18	1 (reference)		<0.001	
Former smoking	18203	181 (0.99)	128,421	1.41	6.19 (4.56–8.41)	<0.001		
Current smoking	6900	183 (2.65)	48,575	3.77	16.85 (12.25–23.19)	<0.001		
Genetic risk and smoking pack-years								
Low genetic risk								0.959
No (0) smoking	44,186	44 (0.10)	314,762	0.14	1 (reference)		<0.001	
Light (0.1–19.9) smoking	13,116	20 (0.15)	93,184	0.21	1.49 (0.88–2.53)	0.142		
Intermediate (20–39.9) smoking	8103	82 (1.01)	57,352	1.43	7.90 (5.39–11.56)	<0.001		
Heavy (≥40) smoking	3750	102 (2.72)	26,087	3.91	16.22 (10.97–23.97)	<0.001		
Intermediate genetic risk								
No (0) smoking	133,546	165 (0.12)	951,246	0.17	1 (reference)		<0.001	
Light (0.1–19.9) smoking	38,157	154 (0.40)	270,270	0.57	3.26 (2.61–4.06)	<0.001		
Intermediate (20–39.9) smoking	24,437	315 (1.29)	172,743	1.82	8.32 (6.85–10.11)	<0.001		
Heavy (≥40) smoking	11,325	385 (3.40)	78,500	4.90	17.06 (13.97–20.84)	<0.001		
High genetic risk								
No (0) smoking	44,277	59 (0.13)	315,218	0.19	1 (reference)		<0.001	
Light (0.1–19.9) smoking	12,596	63 (0.50)	89,236	0.71	3.68 (2.58–5.27)	<0.001		
Intermediate (20–39.9) smoking	8349	130 (1.56)	58,992	2.20	9.89 (7.19–13.59)	<0.001		
Heavy (≥40) smoking	3952	168 (4.25)	27,325	6.15	21.22 (15.34–29.35)	<0.001		

*IR* incidence rate, *HR* hazard ratio, *CI* confidence interval.

^a^Incidence rates are provided per 1000 person-years.

^b^Cox proportional hazards regression adjusted for age, sex, education, Townsend deprivation index, income, BMI, diet, physical activity, alcohol consumption, occupational exposure, passive smoking, relatedness, and first 20 principal components of ancestry. *P* value for trend calculated treating each smoking category as continuous variables.

The same pattern of associations was observed in a series of sensitivity analyses with additional adjustment for COPD and chronic pulmonary infections, excluding participants who had third-degree or higher relatedness, excluding participants who developed outcomes within two years of baseline, and those who had a mismatch between calculation and self-reported never-smoking. (eTables 6 and 7 in the Supplement). Stratified analyses were performed by age and sex (eTables 8 and 9 in the Supplement), but the results were not markedly different among male and female or the <60 years and ≥60 years groups.

### Population-attributable fractions

Since there was no significant interaction between PRS and smoking, the population-attributable fractions were calculated regardless of genetic risk. If all individuals had never smoked, 76.4% (95% CI, 73.4–79.2, based on smoking status) to 75.3% (95% CI, 72.0–78.2, based on smoking pack-years) new-onset lung cancer events might have been prevented during follow-up. If all current smokers quit smoking and the former smokers remained, the new-onset events might have been reduced by 26.4% (95% CI, 25.8–27.0). Further analyses stratified by genetic-risk category showed that 73.4% (95% CI, 64.5–80.4), 76.1% (95% CI, 72.2–79.6), and 79.1% (95% CI, 73.0–83.9) of incident lung cancer cases were attributed to smoking among the low, intermediate and high genetic-risk populations. When the smoking status proportional in HSE and EPIC were included, the PAFs of smoking were 83.2% (95% CI, 80.9–85.3) and 85.1% (95% CI, 83.1–87.0), respectively (eTable 10 in the Supplement).

## Discussion

In this large population-based prospective cohort study of more than 345,000 European individuals, high genetic risk and smoking status were independently associated with an increased risk of incident lung cancer events. Among never-smokers, there was no significant difference in the incident risk between each genetic group. The high genetic risk was two-fold higher than that of low genetic risk for current smokers. A similar pattern was observed for genetic risk and smoking pack-year groups. Meanwhile, there was no significant interaction between the PRS and smoking status or pack-years for incident lung cancer, and smoking cessation or reduction can provide similar protection against lung cancer regardless of genetic risk. The PAF analysis hypothesised that ~76% of new-onset lung cancer events might have been prevented if all individuals had never smoked.

To our knowledge, this study is by far the most extensive and fully adjusted prospective study of lung cancer incidence treating smoking as a single modifiable factor and incorporating multiple genetic-risk factors. Many common variants with minor effects have been identified as associated with a high risk of lung cancer, and the PRS can indicate their combined impact. Previous studies used 19 SNPs to construct a PRS for non-small cell lung cancer and showed predictive effects in a prospective study of 95,408 individuals [9]. Compared with this previous study, the present study included a larger sample size and more SNPs to increase the power for risk estimation. Meanwhile, we used the upper and lower quintiles to categorise the high and low genetic-risk groups [35, 36], which may reduce the accuracy for the high genetic-risk group but warn a broader population that they need to carry out PRS-informed disease screening or life planning for life-threatening lung cancer. It also ensured that the comparison between the combined smoking and genetic-risk subgroups had sufficient statistical power.

Compared with another study based on the UK Biobank [37], the current PRS contains fewer highly independent SNPs in each locus to avoid overinflation of the GWAS summary results caused by many linkage disequilibrium SNPs. Therefore, this PRS may have better generalisations in other populations [38]. The current results showed similar HRs after adjusting for confounding factors (economic and social background, lifestyle factors, occupational exposure). Compared with case-control studies [39, 40], prospective studies may lose some statistical power, but estimates of the absolute risk support using the PRS to predict incident lung cancer [10, 41]. Regarding the role of PRS in never-smokers, our results suggest that their incident risk did not achieve statistical significance as the PRS group increased. Among never-smokers, the post hoc study powers for incident lung cancer in those with intermediate and high genetic risk were only 0.243–0.293. Therefore, we speculate that more outcome events may bring different results with the extension of follow-up time. To sum up, we believe that PRS could be a powerful tool for lung cancer risk assessment as it provides additional information independent of smoking and combining it with traditional risk factors could contribute to a better prediction of lung cancer.

We observed a strong association between smoking and incident lung cancer, independent of genetic risk, and the increased risk was much greater than the genetic risk. This means that smoking will significantly offset low genetic-risk benefits, consistent with a previous study [9]. However, we followed the same grouping method and found that the risk values were much more significant than those in a previous study (eTable 11 in the Supplement). Sample size, confounding factors, subtle differences in smoking habits, and outcome data sources may be the reasons for the differences. We observed similar associations between smoking and lung cancer with other relevant studies [42, 43]. Based on a study of the contemporary population, although smoking, a long-recognised risk factor has undergone tremendous changes in production, composition and use method [44], it still plays a decisive role in lung cancer occurrence. Therefore, smoking cessation is still the most significant and cost-effective way to prevent lung cancer.

Previous studies believed that smoking was responsible for 80%~90% of lung cancer [2, 43, 45], and a study showed that 63.6% of lung cancer are attributable to comprehensive modifiable factors, including smoking and air pollution [37]. We found that the entire population would avoid 76.4% of lung cancer cases by becoming never-smokers. The slight reduction in this proportion is probably because of the reduction in smoking prevalence (23.3% of individuals were current smokers in The European Prospective Investigation into Cancer and Nutrition cohort [43]), manifesting the achievement of tobacco use control. In addition, differences in sample, methodology, and confounders’ representativeness also contribute to the different PAFs between studies. Furthermore, we also estimated the attribution of smoking by a more natural form of PAFs called the generalised impact fraction [46]. Our results showed that if all current smokers stop smoking and former smokers remain, the expected reduction in lung cancer cases would be 26%, again highlighting the efficiency of smoking cessation.

GWASs have shown that a locus may be simultaneously associated with smoking preference and lung cancer [12, 47, 48]. The interaction between smoking and genetic risk for lung cancer is a topic worth discussing, as it may help explain some of the missing heritability in lung cancer susceptibility [49]. Variants at the 15q25 locus have been confirmed by several studies associated with increased tobacco addiction and lung cancer risk [47, 48], but a significant gene-environment interaction is controversial [50, 51]. Some studies suggested that there were significant gene-smoking interactions at 10q25 [52], 14q22, 15q22 [53] and 19q13 [54]. In this study, there was no significant PRS-smoking interaction for lung cancer. This may be because the combination of multiple loci may mask the potential interaction, and the model selection and the specific definition of smoking habits may also affect the results. Besides, the number of positive cases observed in this cohort was far less than in large-scale GWASs, so there may be insufficient statistical power. However, based on the analysis of adjusting for extensive potential confounding factors and using the two smoking measures, we still believe that PRS and smoking promote lung cancer independently.

### Strengths and limitations

This study has several strengths. Many participants from the UK Biobank study provided complete exposure information, and the extensive phenotype information provided many covariates that could be adjusted in the model to eliminate potential confounders. A more detailed grouping of lifetime tobacco exposure showed a typical dose-response relationship. Furthermore, the study population was utterly independent of previous GWASs that identified the risk loci and their effect sizes, which avoided overfitting to some extent.

Several limitations also need to be considered. First, the analysis was conducted on overall lung cancer without constructing PRS and assessing their effects for more detailed lung cancer classifications, which may mask their heterogeneity. Second, additional variants or genetic patterns associated with lung cancer are likely to be identified in the future, which may refine estimates of genetic risk. Third, PRS based on GWASs of European ancestry may limit its application in a larger population due to the differences in risk alleles, allele frequency, and the effect sizes of risk alleles. Fourth, smoking behaviours were self-reported and may have recall and misclassification bias, and there may be differences in the distribution of individuals excluded due to lacking smoking information. Fifth, smoking was not randomly assigned. Although analyses were adjusted for several covariates and sensitivity analyses, the possibility of unmeasured confounding remained. Sixth, the current study included 936 (0.27%) participants with inconsistent information on never-smoking and 0 pack-years of smoking. This may be due to the difference between the self-reported state and participants’ calculated state with minimal smoking exposure. Although we excluded these people in the sensitivity analysis, there may still be potential inconsistencies. Finally, the potential “healthy volunteer” selection bias in the UK biobank may be accompanied by a lower proportion of the smoking population and underestimated PAF. A mild increase in PAF was found using representative England and European population structures.

## Conclusion

In conclusion, high genetic risk and smoking were independently associated with higher lung cancer risk, and there were no interactions between these risk factors. Polygenic risk assessment can provide important information beyond a variety of environmental exposures. This study provided new insights to quantitatively evaluate the role of smoking and genetics in lung cancer.

## Supplementary information

Supplementary Online Content

STROBE Checklist

Reproducibility Checklist

## Data Availability

The dataset supporting the conclusions of this article is available in the UK Biobank upon request (https://www.ukbiobank.ac.uk/).
