# Supplementary material for: Association of smoking and polygenic risk with the incidence of lung cancer: a prospective cohort study
Source: Br J Cancer. 2022 Feb 22;126(11):1637–46. doi: 10.1038/s41416-022-01736-3 (PMC9130319; doi:10.1038/s41416-022-01736-3)
Supplement: Supplementary file 1 — Supplementary Online Content [file 41416_2022_1736_MOESM1_ESM.docx]

Supplementary Online Content

Table of Contents

[**eTable 1. Single-Nucleotide Polymorphisms Used to Build the Genetic Risk Score for Lung Cancer** 1](#_Toc90408377)

[**eTable 2. Smoking definition** 3](#_Toc90408378)

[**eTable 3. Disease definitions used in the UK Biobank study** 4](#_Toc90408379)

[**eTable 4. Risk of Incident Lung Cancer According to Genetic Risk Quintile** 5](#_Toc90408380)

[**eTable 5. Risk of Incident Lung Cancer According to Numbers of Smoking Pack-years** 6](#_Toc90408381)

[**eTable 6. Risk of Incident Lung Cancer According to Genetic Risk and Smoking Status with Additional Adjustment for Respiratory Diseases, After Excluding Related Participants and Excluding Participants with Outcomes Within 2 Years of Baseline** 7](#_Toc90408382)

[**eTable 7. Risk of Incident Lung Cancer According to Genetic Risk and Smoking Pack-years with Additional Adjustment for Respiratory Diseases, After Excluding Related Participants and Excluding Participants with Outcomes Within 2 Years of Baseline** 9](#_Toc90408383)

[**eTable 8. Risk of Incident Lung Cancer According to Genetic Risk and Smoking Status Stratified by Sociodemographic Variables** 11](#_Toc90408384)

[**eTable 9. Risk of Incident Lung Cancer According to Genetic Risk and Smoking Pack-years Stratified by Sociodemographic Variables** 12](#_Toc90408385)

[**eTable 10. Population Attributable Fraction per Smoking Group** 13](#_Toc90408386)

[**eTable 11. Risk of Incident Lung Cancer According to Genetic Risk and Smoking Pack-years Compared with Non-Smokers** 14](#_Toc90408387)

[**eFigure 1. Multivariable Adjusted Hazard Ratios for Incident Lung Cancer According to PRS on a Continuous Scale** 15](#_Toc90408388)

**eTable 1. Single-Nucleotide Polymorphisms Used to Build the Genetic Risk Score for Lung Cancer**

| SNP | Chromosome | Region | Position | Risk allele | Non-risk allele | Trait | Gene(s) | P value | OR | Source |
| --- | --- | --- | --- | --- | --- | --- | --- | --- | --- | --- |
| rs71658797 | 1 | 1p31.1 | 77967507 | A | T | Lung cancer | AK5 | 3.00E-11 | 1.136 | McKay JD, 2017 |
| rs17038564 | 2 | 2p14 | 65496058 | G | A | Lung adenocarcinoma | AFTPH | 2.00E-08 | 1.15 | Dai J, 2019 |
| rs3769821 | 2 | 2q33.1 | 202123430 | C | T | Non-small cell lung cancer | CASP8 | 4.00E-08 | 1.08 | Dai J, 2019 |
| rs2293607 | 3 | 3q26.2 | 169482335 | T | C | Non-small cell lung cancer | MYNN | 2.00E-10 | 1.1 | Dai J, 2019 |
| rs13314271 | 3 | 3q28 | 189357602 | T | C | Lung cancer | TP63 | 7.00E-10 | 1.13 | Wang Y, 2014 |
| rs13167280 | 5 | 5p15.33 | 1280477 | A | G | Non-small cell lung cancer | TERT | 5.00E-32 | 1.24 | Dai J, 2019 |
| rs7705526 | 5 | 5p15.33 | 1285974 | A | C | Lung adenocarcinoma | TERT | 4.00E-35 | 1.249 | McKay JD, 2017 |
| rs4975616 | 5 | 5p15.33 | 1315660 | G | A | Lung cancer | CLPTM1L | 3.00E-09 | 1.15 | Broderick P, 2009 |
| rs1056503 | 5 | 5q14.2 | 82648977 | G | T | Lung cancer | XRCC4 | 6.20E-05 | 1.11 | Wang M, 2016 |
| rs2895680 | 5 | 5q32 | 146644115 | C | T | Lung cancer | PPP2R2B-STK32A-  DPYSL3 | 6.60E-09 | 1.14 | Dong J, 2012 |
| rs2517873 | 6 | 6p22.1 | 29875992 | A | G | Non-small cell lung cancer | MHC | 5.00E-11 | 1.16 | Dai J, 2019 |
| rs3817963 | 6 | 6p22.1 | 32368087 | C | T | Lung adenocarcinoma | BTNL2, TSBP1-AS1 | 1.00E-16 | 1.29 | Shiraishi K, 2012 |
| rs1853837 | 6 | 6p21.1 | 41497035 | A | C | Non-small cell lung cancer | FOXP4 | 1.00E-10 | 1.12 | Dai J, 2019 |
| rs5879422 | 6 | 6q22.1 | 117784658 | T | TTG | Non-small cell lung cancer | DCBLD1 | 4.00E-09 | 1.08 | Dai J, 2019 |
| rs6920364 | 6 | 6q27 | 167376466 | C | G | Lung cancer | RNASET2, MIR3939 | 1.00E-08 | 1.07 | McKay JD, 2017 |
| rs11780471 | 8 | 8p21.2 | 27344719 | G | A | Lung cancer | CHRNA2, EPHX2 | 2.00E-08 | 1.152 | McKay JD, 2017 |
| rs4236709 | 8 | 8p12 | 32410110 | G | A | Non-small cell lung cancer | NRG1 | 1.00E-12 | 1.12 | Dai J, 2019 |
| rs10429489 | 9 | 9p21.3 | 21787521 | A | G | Non-small cell lung cancer | CDKN2A | 7.00E-10 | 1.11 | Dai J, 2019 |
| rs62560775 | 9 | 9p21.3 | 22052068 | G | A | Lung cancer | CDKN2B-AS1 | 6.00E-07 | 1.106 | McKay JD, 2017 |
| rs1333040 | 9 | 9p21.3 | 22083404 | C | T | Lung squamous cell carcinoma | CDKN2B-AS1 | 7.00E-07 | 1.098 | McKay JD, 2017 |
| rs4573350 | 9 | 9q33.2 | 124955115 | T | C | Lung squamous cell carcinoma | DAB2IP | 3.00E-09 | 1.13 | Dai J, 2019 |
| rs1663689 | 10 | 10p14 | 9025195 | T | C | Lung cancer | GATA3 | 2.80E-10 | 1.136 | Dong J, 2012 |
| rs12415204 | 10 | 10q23.33 | 95330890 | A | C | Lung cancer | FFAR4 | 1.60E-04 | 1.09 | Poirier JG, 2015 |
| rs11591710 | 10 | 10q24.33 | 105687632 | C | A | Lung adenocarcinoma | OBFC1, SLK | 6.00E-11 | 1.162 | McKay JD, 2017 |
| rs12265047 | 10 | 10q25.2 | 114487925 | G | A | Non-small cell lung cancer | VTI1A | 8.00E-10 | 1.13 | Dai J, 2019 |
| rs55768116 | 11 | 11q23.3 | 118108331 | C | A | Non-small cell lung cancer | MPZL3 | 2.00E-13 | 1.1 | Dai J, 2019 |
| rs7953330 | 12 | 12p13.33 | 998819 | G | C | Lung cancer | WNK1 | 6.00E-12 | 1.091 | McKay JD, 2017 |
| rs11571833 | 13 | 13q13.1 | 32972626 | T | A | Lung cancer | BRCA2 | 6.00E-16 | 1.603 | McKay JD, 2017 |
| rs1200399 | 14 | 14q13.2 | 35293185 | C | T | Non-small cell lung cancer | BAZ1A | 3.00E-09 | 1.11 | Dai J, 2019 |
| rs66759488 | 15 | 15q21.1 | 47577451 | A | G | Lung cancer | SEMA6D | 3.00E-08 | 1.07 | McKay JD, 2017 |
| rs77468143 | 15 | 15q21.1 | 49376624 | T | G | Lung cancer | SECISBP2L, COPS2 | 1.00E-09 | 1.086 | McKay JD, 2017 |
| rs8034191 | 15 | 15q25.1 | 78806023 | C | T | Lung cancer | CHRNA3 | 3.00E-26 | 1.29 | Broderick P, 2009 |
| rs56113850 | 19 | 19q13.2 | 41353107 | C | T | Lung cancer | CYP2A6 | 5.00E-19 | 1.131 | McKay JD, 2017 |

Abbreviations: SNP: Single Nucleotide Polymorphisms, OR: odds ratio.

**eTable 2. Smoking definition**

|  | Definition | UK Biobank Self Report Fields |
| --- | --- | --- |
| Smoking status | UK Biobank touchscreen questionnaire at baseline: smoking status defined as never, previous, or current smoker | 1239, 1249, 2644, 20116 |
| Smoking pack-years | UK Biobank touchscreen questionnaire at baseline: Pack-years = Number of cigarettes per day / 20 × (Age stopped smoking - Age start smoking) Besides, the data of individuals who quit smoking for more than six months were adjusted. According to the data field 2907 or 3486, subtract six months from the years of smoking. Pack-years = Number of cigarettes per day / 20 × (Age stopped smoking - Age start smoking - 0.5) Note: Individuals who reported starting and stopping smoking at the same age and reported giving up smoking for more than six months had pack-years set at 0. | 2867, 2887, 2897, 2907, 3436, 3486, 6183, 6194, 20161 |

**eTable 3. Disease definitions used in the UK Biobank study**

| Disease | UK Biobank Self Report Fields and Codes | ICD 9 | ICD 10 |
| --- | --- | --- | --- |
| Lung cancer | Field 20001:  Code 1001, Code 1028 | 162, 1620, 1622, 1623, 1624, 1625, 1628, 1629 | C34, C34.0, C34.1, C34.2, C34.3, C34.8, C34.9 |
| COPD | Field 20002:  Code 1112, Code 1113, Code 1472 | 492, 492.0, 492.8, 496.X | J43, J43.0, J43.1, J43.2, J43.8, J43.9, J44, J44.0, J44.1, J44.8, J44.9. |
| Chronic Pulmonary Infections | Field 20002:  Code 1412, Code 1114, Code 1594, Code 1411, Code 1498 | 491, 491.1, 491.8, 491.9, 494, 494.1, 510, 510.9, 511, 513, 513.1, 530.84 | J41.0, J41.1, J41.8, J42, J47.0, J47.1, J47.9, J85.0, J85.1, J85.2, J85.3, J86.0, J86.9 |

Abbreviations: ICD, International Classification of Diseases; COPD, chronic obstructive pulmonary disease.

**eTable 4. Risk of Incident Lung Cancer According to Genetic Risk Quintile**

| Genetic Risk | Total No. of participants | No. of cases (%) | Person-years | IR^a^ | Model 1^b^ | | | Model 2^c^ | | | Model 3^d^ | | |
| --- | --- | --- | --- | --- | --- | --- | --- | --- | --- | --- | --- | --- | --- |
|  |  |  |  |  | HR (95% CI) | P value | P for trend | HR (95% CI) | P value | P for trend | HR (95% CI) | P value | P for trend |
| Quintile 1 (lowest) | 69155 | 248 (0.36) | 491385 | 0.5 | 1 (reference) | | <0.001 | 1 (reference) | | <0.001 | 1 (reference) | | <0.001 |
| Quintile 2 | 69145 | 332 (0.48) | 490792 | 0.68 | 1.35 (1.15 to 1.6) | <0.001 |  | 1.37 (1.16 to 1.61) | <0.001 |  | 1.35 (1.14 to 1.59) | <0.001 |  |
| Quintile 3 | 69150 | 350 (0.51) | 490917 | 0.71 | 1.43 (1.22 to 1.68) | <0.001 |  | 1.43 (1.21 to 1.68) | <0.001 |  | 1.4 (1.19 to 1.65) | <0.001 |  |
| Quintile 4 | 69170 | 337 (0.49) | 491051 | 0.69 | 1.39 (1.18 to 1.64) | <0.001 |  | 1.41 (1.2 to 1.66) | <0.001 |  | 1.38 (1.17 to 1.63) | <0.001 |  |
| Quintile 5 (highest) | 69174 | 420 (0.61) | 490771 | 0.86 | 1.73 (1.48 to 2.03) | <0.001 |  | 1.73 (1.48 to 2.02) | <0.001 |  | 1.69 (1.44 to 1.97) | <0.001 |  |

Abbreviations: IR, incidence rate; HR, hazard ratio; CI, confidence interval.

^a^ Incidence rates are provided per 1000 person-years.

^b^ Model 1: Cox proportional hazards regression adjusted for age, sex, education, Townsend deprivation index, income, BMI, diet, physical activity, alcohol consumption, occupational exposure, passive smoking, relatedness, and first 20 principal components of ancestry; P value for trend calculated treating the polygenic risk score as a continuous variable.

^c^ Model 2: Cox proportional hazards regression adjusted for Model 1 and smoking status categories; P value for trend calculated treating the genetic risk score as a continuous variable.

^d^ Model 3: Cox proportional hazards regression adjusted for Model 1 and smoking pack-years categories; P value for trend calculated treating the genetic risk score as a continuous variable.

**eTable 5. Risk of Incident Lung Cancer According to Numbers of Smoking Pack-years**

| Smoking Pack-years: | Total No. of participants | No. of cases (%) | Person-years | IR^a^ | Model 1^b^ | | | Model 2^c^ | | |
| --- | --- | --- | --- | --- | --- | --- | --- | --- | --- | --- |
|  |  |  |  |  | HR (95% CI) | P value | P for trend | HR (95% CI) | P value | P for trend |
| 0 | 222009 | 268 (0.12) | 1581227 | 0.17 | 1 (reference) | | <0.001 | 1 (reference) | | <0.001 |
| 0.1-9.9 | 30256 | 71 (0.23) | 214549 | 0.33 | 2.01 (1.55 to 2.62) | <0.001 |  | 2.02 (1.55 to 2.62) | <0.001 |  |
| 10-19.9 | 33613 | 166 (0.49) | 238141 | 0.70 | 3.90 (3.21 to 4.74) | <0.001 |  | 3.91 (3.22 to 4.75) | <0.001 |  |
| 20-29.9 | 24292 | 247 (1.02) | 172196 | 1.43 | 7.22 (6.05 to 8.61) | <0.001 |  | 7.22 (6.06 to 8.62) | <0.001 |  |
| 30-39.9 | 16597 | 280 (1.69) | 116890 | 2.40 | 10.67 (8.97 to 12.69) | <0.001 |  | 10.63 (8.93 to 12.64) | <0.001 |  |
| ≥40 | 19027 | 655 (3.44) | 131912 | 4.97 | 18.25 (15.61 to 21.33) | <0.001 |  | 18.16 (15.53 to 21.22) | <0.001 |  |

Abbreviations: IR, incidence rate; HR, hazard ratio; CI, confidence interval.

^a^ Incidence rates are provided per 1000 person-years.

^b^ Model 1: Cox proportional hazards regression adjusted for age, sex, education, Townsend deprivation index, income, BMI, diet, physical activity, alcohol consumption, occupational exposure, passive smoking, relatedness, and first 20 principal components of ancestry; P value for trend calculated treating each smoking categories as continuous variables.

^c^ Model 2: Cox proportional hazards regression adjusted for Model 1 and polygenic risk score; P value for trend calculated treating each smoking categories as continuous variables.

**eTable 6. Risk of Incident Lung Cancer According to Genetic Risk and Smoking Status with Additional Adjustment for Respiratory Diseases, After Excluding Related Participants and Excluding Participants with Outcomes Within 2 Years of Baseline**

| Subgroup | COPD^a^ (n=345794) | | Chronic Pulmonary Infections^b^ (n=345794) | | Excluded related participants^c^ (n=237501) | | Excluded outcomes within two years^d^ (n=344367) | | Excluded never smoking mismatch^d^ (n=344858) | |
| --- | --- | --- | --- | --- | --- | --- | --- | --- | --- | --- |
|  | HR (95% CI) | P value | HR (95% CI) | P value | HR (95% CI) | P value | HR (95% CI) | P value | HR (95% CI) | P value |
| Low genetic risk |  |  |  |  |  |  |  |  |  |  |
| Never smoking | 1 (reference) | | 1 (reference) | | 1 (reference) | | 1 (reference) | | 1 (reference) | |
| Former smoking | 4.8 (3.36 to 6.86) | <0.001 | 4.74 (3.32 to 6.78) | <0.001 | 4.31 (2.82 to 6.58) | <0.001 | 5.24 (3.52 to 7.81) | <0.001 | 4.88 (3.42 to 6.97) | <0.001 |
| Current smoking | 11.12 (7.71 to 16.06) | <0.001 | 10.83 (7.5 to 15.64) | <0.001 | 8.48 (5.41 to 13.29) | <0.001 | 12.34 (8.2 to 18.56) | <0.001 | 11.32 (7.84 to 16.35) | <0.001 |
| Intermediate genetic risk |  |  |  |  |  |  |  |  |  |  |
| Never smoking | 1.23 (0.88 to 1.73) | 0.232 | 1.23 (0.87 to 1.73) | 0.235 | 1.15 (0.77 to 1.71) | 0.495 | 1.30 (0.88 to 1.90) | 0.183 | 1.23 (0.88 to 1.73) | 0.232 |
| Former smoking | 6.28 (4.57 to 8.64) | <0.001 | 6.16 (4.48 to 8.47) | <0.001 | 5.59 (3.85 to 8.12) | <0.001 | 6.49 (4.53 to 9.29) | <0.001 | 6.38 (4.64 to 8.77) | <0.001 |
| Current smoking | 17.63 (12.78 to 24.31) | <0.001 | 17.2 (12.47 to 23.72) | <0.001 | 16.32 (11.2 to 23.78) | <0.001 | 17.93 (12.48 to 25.76) | <0.001 | 17.95 (13.02 to 24.76) | <0.001 |
| High genetic risk |  |  |  |  |  |  |  |  |  |  |
| Never smoking | 1.33 (0.89 to 1.98) | 0.163 | 1.33 (0.89 to 1.98) | 0.165 | 1.35 (0.85 to 2.16) | 0.200 | 1.18 (0.74 to 1.88) | 0.479 | 1.33 (0.89 to 1.98) | 0.162 |
| Former smoking | 8.03 (5.73 to 11.25) | <0.001 | 7.91 (5.65 to 11.09) | <0.001 | 7.77 (5.23 to 11.54) | <0.001 | 7.93 (5.42 to 11.61) | <0.001 | 8.20 (5.85 to 11.48) | <0.001 |
| Current smoking | 22.06 (15.71 to 30.99) | <0.001 | 21.38 (15.22 to 30.04) | <0.001 | 21.74 (14.59 to 32.39) | <0.001 | 25.00 (17.11 to 36.54) | <0.001 | 22.34 (15.9 to 31.39) | <0.001 |

Abbreviations: HR, hazard ratio; CI, confidence interval; COPD, chronic obstructive pulmonary disease.

^a^ Adjusted for age, sex, education, Townsend deprivation index, income, BMI, diet, physical activity, alcohol consumption, occupational exposure, passive smoking, relatedness, COPD, and first 20 principal components of ancestry.

^b^ Adjusted for age, sex, education, Townsend deprivation index, income, BMI, diet, physical activity, alcohol consumption, occupational exposure, passive smoking, relatedness, chronic pulmonary infections, and first 20 principal components of ancestry.

^c^ Adjusted for age, sex, education, Townsend deprivation index, income, BMI, diet, physical activity, alcohol consumption, occupational exposure, passive smoking, and first 20 principal components of ancestry.

^d^ Adjusted for age, sex, education, Townsend deprivation index, income, BMI, diet, physical activity, alcohol consumption, occupational exposure, passive smoking, relatedness, and first 20 principal components of ancestry.

**eTable 7. Risk of Incident Lung Cancer According to Genetic Risk and Smoking Pack-years with Additional Adjustment for Respiratory Diseases, After Excluding Related Participants and Excluding Participants with Outcomes Within 2 Years of Baseline**

| Subgroup | COPD^a^ (n=345794) | | Chronic Pulmonary Infections^b^ (n=345794) | | Excluded related participants^c^ (n=237501) | | Excluded outcomes within two years^d^ (n=344367) | | Excluded never smoking mismatch^d^ (n=344858) | |
| --- | --- | --- | --- | --- | --- | --- | --- | --- | --- | --- |
|  | HR (95% CI) | P value | HR (95% CI) | P value | HR (95% CI) | P value | HR (95% CI) | P value | HR (95% CI) | P value |
| Low genetic risk |  |  |  |  |  |  |  |  |  |  |
| No (0) smoking | 1 (reference) | | 1 (reference) | | 1 (reference) | | 1 (reference) | | 1 (reference) | |
| Light (0.1-19.9) smoking | 1.49 (0.88 to 2.53) | 0.137 | 1.49 (0.88 to 2.52) | 0.143 | 0.99 (0.49 to 2.01) | 0.983 | 1.58 (0.89 to 2.83) | 0.121 | 1.55 (0.91 to 2.65) | 0.106 |
| Intermediate (20-39.9) smoking | 7.98 (5.52 to 11.54) | <0.001 | 7.91 (5.47 to 11.43) | <0.001 | 6.64 (4.26 to 10.33) | <0.001 | 8.44 (5.61 to 12.7) | <0.001 | 8.3 (5.71 to 12.07) | <0.001 |
| Heavy (≥40) smoking | 16.5 (11.51 to 23.64) | <0.001 | 15.93 (11.12 to 22.84) | <0.001 | 13.64 (8.85 to 21.00) | <0.001 | 17.05 (11.44 to 25.43) | <0.001 | 16.82 (11.67 to 24.25) | <0.001 |
| Intermediate genetic risk |  |  |  |  |  |  |  |  |  |  |
| No (0) smoking | 1.24 (0.89 to 1.73) | 0.206 | 1.24 (0.89 to 1.73) | 0.207 | 1.15 (0.78 to 1.69) | 0.487 | 1.28 (0.89 to 1.86) | 0.186 | 1.23 (0.87 to 1.72) | 0.243 |
| Light (0.1-19.9) smoking | 4 (2.86 to 5.59) | <0.001 | 3.97 (2.84 to 5.55) | <0.001 | 3.3 (2.22 to 4.91) | <0.001 | 4.07 (2.8 to 5.93) | <0.001 | 4.15 (2.95 to 5.84) | <0.001 |
| Intermediate (20-39.9) smoking | 10.26 (7.47 to 14.1) | <0.001 | 10.1 (7.35 to 13.87) | <0.001 | 8.65 (5.96 to 12.55) | <0.001 | 9.85 (6.89 to 14.07) | <0.001 | 10.59 (7.66 to 14.65) | <0.001 |
| Heavy (≥40) smoking | 20.73 (15.07 to 28.51) | <0.001 | 20 (14.53 to 27.51) | <0.001 | 19.48 (13.43 to 28.25) | <0.001 | 20.03 (14.01 to 28.64) | <0.001 | 21.1 (15.24 to 29.22) | <0.001 |
| High genetic risk |  |  |  |  |  |  |  |  |  |  |
| No (0) smoking | 1.33 (0.9 to 1.97) | 0.150 | 1.33 (0.9 to 1.97) | 0.150 | 1.33 (0.85 to 2.09) | 0.218 | 1.2 (0.76 to 1.87) | 0.436 | 1.32 (0.89 to 1.98) | 0.170 |
| Light (0.1-19.9) smoking | 4.91 (3.34 to 7.22) | <0.001 | 4.89 (3.32 to 7.18) | <0.001 | 4.43 (2.81 to 7) | <0.001 | 5.07 (3.3 to 7.78) | <0.001 | 5.1 (3.45 to 7.54) | <0.001 |
| Intermediate (20-39.9) smoking | 12.8 (9.08 to 18.06) | <0.001 | 12.65 (8.97 to 17.85) | <0.001 | 13.07 (8.77 to 19.46) | <0.001 | 13.3 (9.07 to 19.52) | <0.001 | 13.27 (9.35 to 18.83) | <0.001 |
| Heavy (≥40) smoking | 26.42 (18.84 to 37.05) | <0.001 | 25.37 (18.09 to 35.59) | <0.001 | 24.12 (16.21 to 35.91) | <0.001 | 25.73 (17.62 to 37.59) | <0.001 | 26.77 (18.96 to 37.78) | <0.001 |

Abbreviations: HR, hazard ratio; CI, confidence interval; COPD, chronic obstructive pulmonary disease.

^a^ Adjusted for age, sex, education, Townsend deprivation index, income, BMI, diet, physical activity, alcohol consumption, occupational exposure, passive smoking, relatedness, COPD, and first 20 principal components of ancestry.

^b^ Adjusted for age, sex, education, Townsend deprivation index, income, BMI, diet, physical activity, alcohol consumption, occupational exposure, passive smoking, relatedness, chronic pulmonary infections, and first 20 principal components of ancestry.

^c^ Adjusted for age, sex, education, Townsend deprivation index, income, BMI, diet, physical activity, alcohol consumption, occupational exposure, passive smoking, and first 20 principal components of ancestry.

^d^ Adjusted for age, sex, education, Townsend deprivation index, income, BMI, diet, physical activity, alcohol consumption, occupational exposure, passive smoking, relatedness, and first 20 principal components of ancestry.

**eTable 8. Risk of Incident Lung Cancer According to Genetic Risk and Smoking Status Stratified by Sociodemographic Variables**

| Subgroup | Female^a^ (n=186330) | | Male^a^ (n=159464) | | Age<60 years^b^ (n=200857) | | Age≥60 years^b^ (n=144937) | |
| --- | --- | --- | --- | --- | --- | --- | --- | --- |
|  | HR (95% CI) | P value | HR (95% CI) | P value | HR (95% CI) | P value | HR (95% CI) | P value |
| Low genetic risk |  |  |  |  |  |  |  |  |
| Never smoking | 1 (reference) | | 1 (reference) | | 1 (reference) | | 1 (reference) | |
| Former smoking | 4.02 (2.41 to 6.70) | <0.001 | 5.32 (3.17 to 8.92) | <0.001 | 5.98 (2.72 to 13.15) | <0.001 | 4.42 (2.96 to 6.59) | <0.001 |
| Current smoking | 12.49 (7.55 to 20.66) | <0.001 | 10.29 (5.99 to 17.7) | <0.001 | 12.81 (5.98 to 27.43) | <0.001 | 10.96 (7.2 to 16.69) | <0.001 |
| Intermediate genetic risk |  |  |  |  |  |  |  |  |
| Never smoking | 1.40 (0.90 to 2.19) | 0.137 | 1.00 (0.59 to 1.71) | 0.999 | 2.00 (0.99 to 4.06) | 0.053 | 1.02 (0.69 to 1.51) | 0.928 |
| Former smoking | 7.12 (4.65 to 10.89) | <0.001 | 5.67 (3.51 to 9.18) | <0.001 | 8.05 (4.04 to 16.04) | <0.001 | 5.73 (4.00 to 8.20) | <0.001 |
| Current smoking | 16.88 (10.95 to 26.03) | <0.001 | 18.48 (11.39 to 29.96) | <0.001 | 27.69 (14.05 to 54.58) | <0.001 | 15.06 (10.43 to 21.73) | <0.001 |
| High genetic risk |  |  |  |  |  |  |  |  |
| Never smoking | 1.61 (0.97 to 2.67) | 0.067 | 0.95 (0.49 to 1.85) | 0.888 | 2.20 (1.00 to 4.83) | 0.05 | 1.09 (0.68 to 1.75) | 0.711 |
| Former smoking | 8.90 (5.64 to 14.02) | <0.001 | 7.31 (4.41 to 12.11) | <0.001 | 11.38 (5.49 to 23.57) | <0.001 | 7.09 (4.85 to 10.37) | <0.001 |
| Current smoking | 21.00 (13.18 to 33.46) | <0.001 | 23.58 (14.24 to 39.05) | <0.001 | 32.80 (16.24 to 66.26) | <0.001 | 19.47 (13.16 to 28.81) | <0.001 |

Abbreviations: HR, hazard ratio; CI, confidence interval.

^a^ Adjusted for age, education, Townsend deprivation index, income, BMI, diet, physical activity, alcohol consumption, occupational exposure, passive smoking, relatedness, and first 20 principal components of ancestry.

^b^ Adjusted for age, sex, education, Townsend deprivation index, income, BMI, diet, physical activity, alcohol consumption, occupational exposure, passive smoking, relatedness, and first 20 principal components of ancestry.

**eTable 9. Risk of Incident Lung Cancer According to Genetic Risk and Smoking Pack-years Stratified by Sociodemographic Variables**

| Subgroup | Female^a^ (n=186330) | | Male^a^ (n=159464) | | Age<60 years^b^ (n=200857) | | Age≥60 years^b^ (n=144937) | |
| --- | --- | --- | --- | --- | --- | --- | --- | --- |
|  | HR (95% CI) | P value | HR (95% CI) | P value | HR (95% CI) | P value | HR (95% CI) | P value |
| Low genetic risk |  |  |  |  |  |  |  |  |
| No (0) smoking | 1 (reference) | | 1 (reference) | | 1 (reference) | | 1 (reference) | |
| Light (0.1-19.9) smoking | 1.87 (0.93 to 3.73) | 0.078 | 1.08 (0.48 to 2.46) | 0.851 | 1.26 (0.40 to 3.96) | 0.691 | 1.52 (0.84 to 2.77) | 0.167 |
| Intermediate (20-39.9) smoking | 9.00 (5.38 to 15.06) | <0.001 | 6.60 (3.88 to 11.22) | <0.001 | 11.36 (5.56 to 23.21) | <0.001 | 6.70 (4.35 to 10.31) | <0.001 |
| Heavy (≥40) smoking | 16.55 (9.66 to 28.34) | <0.001 | 13.69 (8.27 to 22.67) | <0.001 | 16.88 (7.73 to 36.87) | <0.001 | 14.23 (9.46 to 21.41) | <0.001 |
| Intermediate genetic risk |  |  |  |  |  |  |  |  |
| No (0) smoking | 1.45 (0.93 to 2.26) | 0.101 | 0.98 (0.59 to 1.63) | 0.943 | 1.70 (0.89 to 3.23) | 0.109 | 1.09 (0.73 to 1.60) | 0.681 |
| Light (0.1-19.9) smoking | 4.57 (2.90 to 7.20) | <0.001 | 3.26 (1.98 to 5.37) | <0.001 | 4.36 (2.24 to 8.50) | <0.001 | 3.78 (2.56 to 5.58) | <0.001 |
| Intermediate (20-39.9) smoking | 11.15 (7.21 to 17.23) | <0.001 | 8.68 (5.45 to 13.85) | <0.001 | 18.06 (9.68 to 33.69) | <0.001 | 7.64 (5.27 to 11.08) | <0.001 |
| Heavy (≥40) smoking | 24.24 (15.56 to 37.76) | <0.001 | 15.85 (9.97 to 25.20) | <0.001 | 30.51 (16.04 to 58.03) | <0.001 | 16.42 (11.38 to 23.7) | <0.001 |
| High genetic risk |  |  |  |  |  |  |  |  |
| No (0) smoking | 1.64 (0.99 to 2.72) | 0.055 | 0.95 (0.51 to 1.79) | 0.885 | 1.79 (0.86 to 3.74) | 0.120 | 1.18 (0.74 to 1.88) | 0.485 |
| Light (0.1-19.9) smoking | 5.36 (3.17 to 9.04) | <0.001 | 4.25 (2.40 to 7.52) | <0.001 | 6.61 (3.17 to 13.82) | <0.001 | 4.29 (2.72 to 6.76) | <0.001 |
| Intermediate (20-39.9) smoking | 14.62 (9.10 to 23.47) | <0.001 | 10.42 (6.31 to 17.22) | <0.001 | 22.58 (11.70 to 43.60) | <0.001 | 9.51 (6.33 to 14.30) | <0.001 |
| Heavy (≥40) smoking | 30.33 (18.74 to 49.07) | <0.001 | 20.49 (12.62 to 33.27) | <0.001 | 34.59 (17.31 to 69.13) | <0.001 | 21.44 (14.54 to 31.60) | <0.001 |

Abbreviations: HR, hazard ratio; CI, confidence interval.

^a^ Adjusted for age, education, Townsend deprivation index, income, BMI, diet, physical activity, alcohol consumption, occupational exposure, passive smoking, relatedness, and first 20 principal components of ancestry.

^b^ Adjusted for age, sex, education, Townsend deprivation index, income, BMI, diet, physical activity, alcohol consumption, occupational exposure, passive smoking, relatedness, and first 20 principal components of ancestry.

**eTable 10. Population Attributable Fraction per Smoking Group**

|  | Whole population | | Low Genetic Risk | | Intermediate Genetic Risk | | High Genetic Risk | | HSE 2010 | | EPIC | |
| --- | --- | --- | --- | --- | --- | --- | --- | --- | --- | --- | --- | --- |
|  | PAF (%) | (95% CI) | PAF (%) | (95% CI) | PAF (%) | (95% CI) | PAF (%) | (95% CI) | PAF (%) | (95% CI) | PAF (%) | (95% CI) |
| Smoking status |  |  |  |  |  |  |  |  |  |  |  |  |
| Former and current smoking to never smoking | 76.4 | (73.4 to 79.2) | 73.4 | (64.5 to 80.4) | 76.1 | (72.2 to 79.6) | 79.1 | (73.0 to 83.9) | 83.2 | (80.9 to 85.3) | 85.1 | (83.1 to 87.0) |
| Current smoking to former smoking | 26.4 | (25.8 to 27.0) | 21.3 | (19.6 to 22.6) | 27.4 | (26.6 to 28.1) | 27.2 | (26.1 to 28.2) | 38.5 | (37.9 to 38.9) | 39.5 | (39.1 to 39.9) |
| Smoking pack-years |  |  |  |  |  |  |  |  |  |  |  |  |
| Smoking to no smoking (0 pack-years) | 75.3 | (72.0 to 78.2) | 72.2 | (62.7 to 79.8) | 74.8 | (70.6 to 78.6) | 78.1 | (71.5 to 83.3) | - | - | - | - |
| Reduce the pack-years of smoking by two level | 72.4 | (69.6 to 75.0) | 71.4 | (62.9 to 78.1) | 71.7 | (68.0 to 75.0) | 74.6 | (68.9 to 79.1) | - | - | - | - |
| Reduce the pack-years of smoking by one level | 56.0 | (54.1 to 57.7) | 56.7 | (50.8 to 61.1) | 55.2 | (52.7 to 57.4) | 57.4 | (53.6 to 60.3) | - | - | - | - |

Abbreviations: PAF, Population Attributable Fraction; CI, Confidence Interval; HSE, Health Survey for England; EPIC, European Prospective Investigation into Cancer and Nutrition.

**eTable 11. Risk of Incident Lung Cancer According to Genetic Risk and Smoking Pack-years Compared with Non-Smokers**

| Subgroup | Total No. of participants | No. of lung cancer cases (%) | Person-years | IR^a^ | HR (95% CI) ^b^ | P value |
| --- | --- | --- | --- | --- | --- | --- |
| No (0) smoking | 222009 | 268 (0.12) | 1581227 | 0.17 | 1 (reference) | |
| Light (<30) smoking |  |  |  |  |  |  |
| Low genetic risk | 4480 | 14 (0.31) | 31829 | 0.44 | 2.38 (1.39 to 4.07) | 0.002 |
| Intermediate genetic risk | 79345 | 434 (0.55) | 562404 | 0.77 | 4.22 (3.62 to 4.93) | <0.001 |
| High genetic risk | 4336 | 36 (0.83) | 30652 | 1.17 | 6.53 (4.61 to 9.26) | <0.001 |
| Heavy (≥30) smoking |  |  |  |  |  |  |
| Low genetic risk | 1754 | 40 (2.28) | 12312 | 3.25 | 11.80 (8.42 to 16.54) | <0.001 |
| Intermediate genetic risk | 31962 | 835 (2.61) | 223165 | 3.74 | 14.01 (12.08 to 16.24) | <0.001 |
| High genetic risk | 1908 | 60 (3.14) | 13326 | 4.50 | 17.24 (12.96 to 22.93) | <0.001 |

Abbreviations: IR, incidence rate; HR, hazard ratio; CI, confidence interval.

^a^ Incidence rates are provided per 1000 person-years.

^b^ Cox proportional hazards regression adjusted for age, sex, education, Townsend deprivation index, income, BMI, diet, physical activity, alcohol consumption, occupational exposure, passive smoking, relatedness, and first 20 principal components of ancestry.


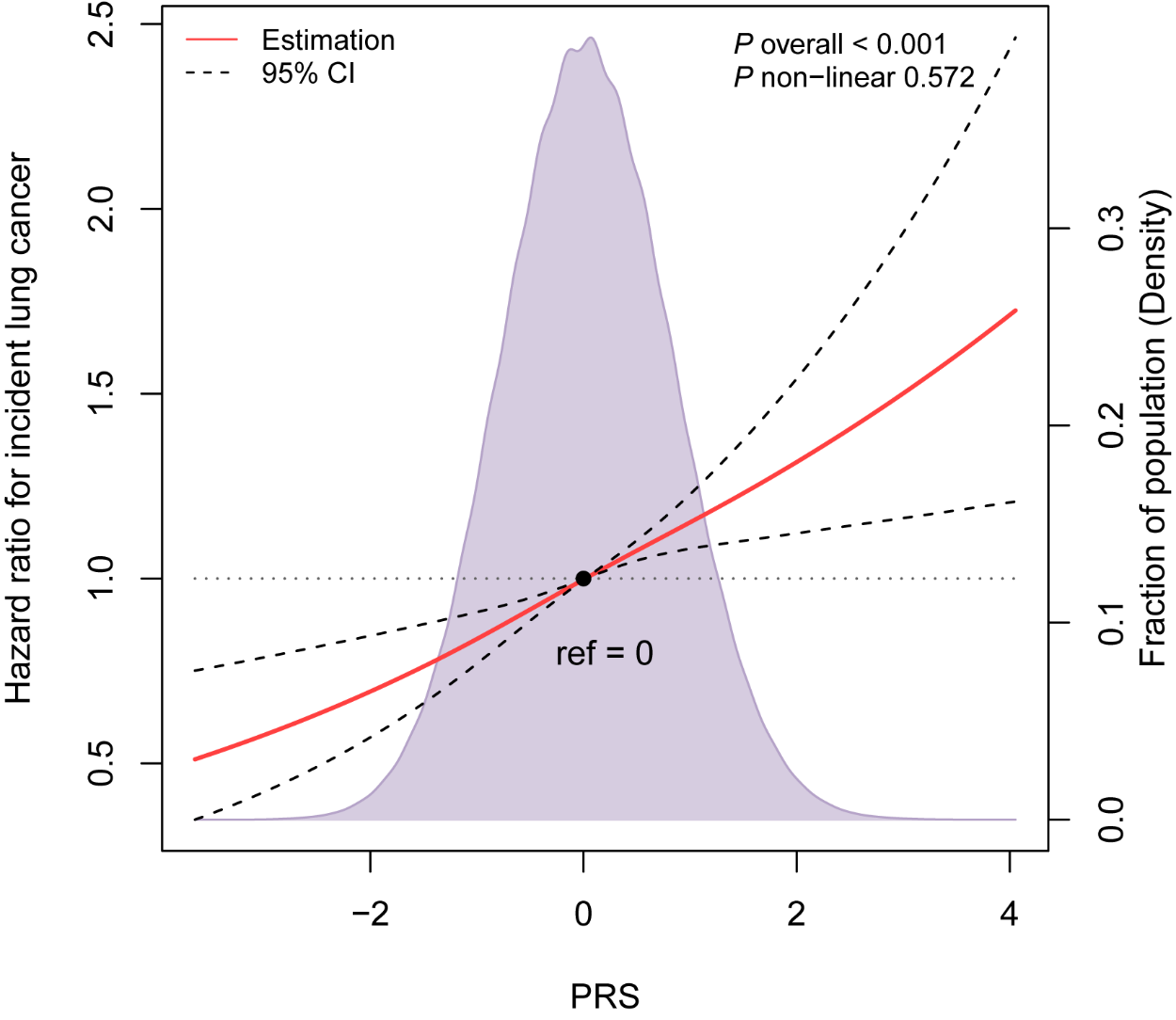


**eFigure 1. Multivariable Adjusted Hazard Ratios for Incident Lung Cancer According to PRS on a Continuous Scale**

Solid red lines are multivariable-adjusted hazard ratios, with dashed black lines showing 95% confidence intervals derived from restricted cubic spline regressions with three knots. Reference lines for no association are indicated by the horizontal lines at a hazard ratio of 1. Solid purple curves show the fraction of the population with different exposures.

Abbreviations: CI, confidence interval; PRS, polygenic risk score.
